# Korovkin-Type Theorems in Weighted *L*_*p*_-Spaces via Summation Process

**DOI:** 10.1155/2013/534054

**Published:** 2013-11-05

**Authors:** Tuncer Acar, Fadime Dirik

**Affiliations:** ^1^Kırıkkale University, Faculty of Science and Arts, Department of Mathematics, Yahşihan, Kırıkkale, Turkey; ^2^Sinop University, Faculty of Sciences and Arts, Department of Mathematics, 57000 Sinop, Turkey

## Abstract

Korovkin-type theorem which is one of the fundamental methods in approximation theory to describe uniform convergence of any sequence of positive linear operators is discussed on weighted *L*_*p*_ spaces, 1 ≤ *p* < *∞* for univariate and multivariate functions, respectively. Furthermore, we obtain these types of approximation theorems by means of *𝒜*-summability which is a stronger convergence method than ordinary convergence.

## 1. Introduction

The fundamental theorem of Korovkin [1] on approximation of continuous functions on a compact interval gives conditions in order to decide whether a sequence of positive linear operators converges to identity operator. This theorem has been extended in several directions. One of the most important papers on these extensions is [2] that where the author obtained Korovkin-type theorem on unbounded sets for the weighted continuous functions on semireal axis. Korovkin-type theorems were also studied on *L*_*p*_-spaces (see [3, 4]).

The extension of Korovkin's theorem from compact intervals to unbounded intervals for functions that belong to *L*_*p*_-spaces was obtained by Gadjiev and Aral [5]. We recall some notations presented in that paper. Let ℝ denote the set of real numbers. The function *ω* is called a weight function if it is positive continuous function on the whole real axis and, for a fixed *p* ∈ [1, *∞*), satisfying the condition

(1) $$\begin{array}{c} \int_{\mathbb{R}}t^{2p}\omega \left(t\right)dt<\infty . \end{array}$$

Let *L*_*p*,*ω*_(ℝ) (1 ≤ *p* < *∞*) denote the linear space of measurable, *p*-absolutely integrable functions on ℝ with respect to weight function *ω*; that is,

(2) Lp,ω(ℝ) ={f:ℝ→ℝ;  ||f||p,ω=(∫ℝ|f(t)|pω(t)dt)1/p<∞}.

The analogues of (1) and (2) in multidimensional space are given as follows. Let *Ω* be a positive continuous function in ℝ^*n*^, satisfying the condition

(3) $$\begin{array}{c} \int_{{\mathbb{R}}^{n}}{\left|t\right|}^{2p}\Omega \left(t\right)dt<\infty , \end{array}$$

and for 1 ≤ *p* < *∞* one has

(4) Lp,Ω(ℝn) ={f:ℝn→ℝ;  ||f||p,Ω=(∫ℝn|f(t)|pΩ(t)dt)1/p<∞}.

The authors obtained Korovkin-type theorems for the functions in *L*_*p*,*ω*_(ℝ) and also in *L*_*p*,*Ω*_(ℝ^*n*^). The aforementioned results are the extensions of Korovkin's theorem on unbounded sets and more general functions spaces by ordinary convergence.

On the other hand, most of the classical operators tend to converge to the value of the function being approximated. At the points of discontinuity, they often converge to the average of the left and right limits of the function. However, there are exceptions which do not converge at points of discontinuity (see [6]). In this case matrix summability methods of Cesáro type are strong enough to correct the lack of convergence [7].

Let *𝒜* : = {*A* ^*n*^} = (*a*_*kj*_^(*n*)^) be a sequence of infinite matrices with non-negative real entries. For a sequence *x* = (*x*_*j*_), the double sequence

(5) $$\begin{array}{c} 𝒜x:=\left\{{\left(Ax\right)}_{k}^{n}:k,\;\;n\in \mathbb{N}\right\}, \end{array}$$

defined by

(6) $$\begin{array}{c} {\left(Ax\right)}_{k}^{n}:=\sum_{j=1}^{\infty }a_{kj}^{\left(n\right)}x_{j}, \end{array}$$

is called *𝒜*-transform of *x* whenever the series that converges for all *k*, *n*, and *x* is said to be *𝒜*-summable to *l* if

(7) $$\begin{array}{c} \underset{k\to \infty }{\lim}\sum_{j=1}^{\infty }a_{kj}^{\left(n\right)}x_{j}=l, \end{array}$$

uniformly in *n* ([8, 9]). If *A* ^*n*^ = *A* for some matrices *A*, then *𝒜*-summability is the ordinary matrix summability by *A*, and if *a*_*kj*_^(*n*)^ = 1/*k*, for *n* ≤ *j* ≤ *k* + *n* (*n* = 1,2, 3,…), and *a*_*kj*_^(*n*)^ = 0 otherwise, then *𝒜*-summability reduces to almost convergence [10]. Replacing the ordinary convergence by *𝒜*-summability some approximation results have been studied in [11–13] and in the special cases [14, 15]. Also, Korovkin-type theorems in weighted space via *𝒜*-summability have been studied in [16, 17].

Our purpose in the present paper is to obtain Korovkin-type theorems on weighted *L*_*p*_ spaces in univariate and multivariate case via *𝒜*-summation process. More precisely, a sequence {*L*_*j*_} of positive linear operators from *L*_*p*,*ω*_ into *L*_*p*,*ω*_ is called an *𝒜*-summation process on *L*_*p*,*ω*_ if (*L*_*j*_(*f*)) is *𝒜*-summable to *f* for every *f* ∈ *L*_*p*,*ω*_; that is,

(8) $$\begin{array}{c} \underset{k}{\lim}{||\sum_{j=1}^{\infty }a_{kj}^{\left(n\right)}L_{j}\left(f\right)-f||}_{p,\omega }=0,\;\text{uniformly}\;\;\text{in}\;\;n, \end{array}$$

where it is assumed that the series in (8) converges for each *k*, *n*, and *f*. Considering this fact we extend (8) to space of sequences of linear positive operators to approximate the functions that belong to *L*_*p*,*ω*_ spaces via matrix summability method. 

## 2. Main Result

Throughout this section we will use the following notations: *A* ^(*n*)^(*f*; *x*) is the double sequence:

(9) $$\begin{array}{c} A_{k}^{\left(n\right)}\left(f;x\right)=\sum_{j=1}^{\infty }a_{kj}^{\left(n\right)}L_{j}\left(f\left(t\right);x\right),\;n=1,2,\ldots , \end{array}$$

and minimum and maximum values of the weight function *ω* on finite intervals will be denoted by *ω*_min⁡_ and *ω*_max⁡_, respectively.

Now we present the following main result.

**Theorem 1**

Let *𝒜* = {*A* ^*n*^} be a sequence of infinite matrices with nonnegative real entries and let {*L*_*j*_} be a sequence of positive linear operators from *L*_*p*,*ω*_ into *L*_*p*,*ω*_. Assume that

(10) $$\begin{array}{c} \underset{n,k}{\sup}{||A_{k}^{\left(n\right)}||}_{L_{p,\omega }\to L_{p,\omega }}<\infty . \end{array}$$

If

(11) $$\begin{array}{c} \underset{k}{\lim}\;\underset{n}{\sup}{||A_{k}^{\left(n\right)}\left(t^{i};x\right)-x^{i}||}_{p,\omega }=0,\;i=0,1,2, \end{array}$$

then for any function *f* ∈ *L*_*p*,*ω*_(ℝ), one has

(12) $$\begin{array}{c} \underset{k}{\lim}\;\underset{n}{\sup}{||A_{k}^{\left(n\right)}f-f||}_{p,\omega }=0. \end{array}$$

**Proof**

Let *χ*_1_^*A*^(*t*) be the characteristic function of the interval [−*A*, *A*] and *χ*_2_^*A*^(*t*) = 1 − *χ*_1_^*A*^(*t*) for any *A* ≥ 0. We can choose a sufficient large *A* such that for every *ε* > 0

(13) $$\begin{array}{c} {||f\chi_{2}^{A}||}_{p,\omega }<\varepsilon . \end{array}$$

Using the assumption of the convergence of the series (9) for each *k*, *n*, and *f* and the linearity of the operators *L*_*j*_, we get

(14) sup⁡n||Ak(n)f−f||p,ω =sup⁡n||Ak(n)(χ1A+χ2A)f−(χ1A+χ2A)f||p,ω ≤sup⁡n||Ak(n)(χ1Af)−χ1Af||p,ω  +sup⁡n||Ak(n)(χ2Af)−χ2Af||p,ω =sup⁡nIn′+sup⁡nIn′′.

By condition (10), there exists a constant *K* > 0 such that

(15) $$\begin{array}{c} \underset{n,k}{\sup}{||A_{k}^{\left(n\right)}||}_{p,\omega }\leq K. \end{array}$$

Hence, from (13), we compute

(16) sup⁡nIn′′≤sup⁡n||Ak(n)χ2Af||p,ω+||χ2Af||p,ω≤(K+1)||χ2Af||p,ω<(K+1)ε.

For every function *f* ∈ *L*_*p*,*ω*_(ℝ) the inequality

(17) $$\begin{array}{c} {||\chi_{2}^{A}f||}_{p}\leq \omega_{\min}^{-1/p}{||f||}_{p,\omega } \end{array}$$

implies that *L*_*p*,*ω*_(−*A*, *A*) ⊂ *L*_*p*_(−*A*, *A*). Since the space of continuous functions is dense in *L*_*p*_(−*A*, *A*), given *f* ∈ *L*_*p*,*ω*_(ℝ), for each *ε*′ > 0, there exists a continuous function *φ* on [−*A*, *A*] satisfying the condition *φ*(*x*) = 0 for |*x*| > *A* such that

(18) $$\begin{array}{c} {||\left(f-\varphi \right)\chi_{1}^{A}||}_{p}<\frac{\varepsilon^{\prime }}{\left(K+1\right)\omega_{\max}^{1/p}}. \end{array}$$

Using the inequalities (15) and (18), we get

(19) sup⁡n In′=sup⁡n||Ak(n)(χ1Af)−χ1Af||p,ω≤sup⁡n||Ak(n)(f−φ)χ1A||p,ω +sup⁡n||Ak(n)(φχ1A)−φχ1A||p,ω+||(f−φ)χ1A||p,ω≤sup⁡n||Ak(n)(φχ1A)−φχ1A||p,ω+ε′.

On the other hand, since *χ*_2_^*A*_1_^ *χ*_1_^*A*^ *φ* = 0 for some *A*_1_ > *A*, we get the inequality

(20) sup⁡n||Ak(n)(φχ1A)−φχ1A||p,ω =sup⁡n||(χ1A1+χ2A1)Ak(n)(φχ1A)−(χ1A1+χ2A1)φχ1A||p,ω ≤sup⁡n||[Ak(n)(φχ1A)−φχ1A]χ1A1||p,ω  +sup⁡n||χ2A1Ak(n)(φχ1A)||p,ω.

Now, by supposing that *M*_*φ*_ = max⁡_*t*∈ℝ_|*φ*(*t*)|*χ*_1_^*A*^(*t*), we get

(21) sup⁡n||χ2A1Ak(n)(φχ1A)||p,ω =sup⁡n(∫|t|>A1|Ak(n)(φχ1A;t)|pω(t)dt)1/p ≤Mφsup⁡n(∫|t|>A1|Ak(n)(1;t)−1|pω(t)dt)1/p  +Mφ(∫ℝχ2A1ω(t)dt)1/p.

Since *ω* ∈ *L*_1_(ℝ), we can choose the number *A*_1_ such that

(22) $$\begin{array}{c} {\left(\int_{\mathbb{R}}^{}\chi_{2}^{A_{1}}\omega \left(t\right)dt\right)}^{1/p}<\frac{\varepsilon^{\prime }}{M_{\varphi }}. \end{array}$$

Using this inequality, we have

(23) $$\begin{array}{c} \underset{n}{\sup}{||\chi_{2}^{A_{1}}A_{k}^{\left(n\right)}\left(\varphi \chi_{1}^{A}\right)||}_{p,\omega }\leq M_{\varphi }\underset{n}{\sup}{||A_{k}^{\left(n\right)}\left(1;x\right)-1||}_{p,\omega }+\varepsilon^{\prime }. \end{array}$$

As a corollary, we get the following inequality for sup⁡_*n*_ *I*_*n*_′:

(24) sup⁡n In′≤2ε′+Mφsup⁡n||Ak(n)(1;x)−1||p,ω+sup⁡n||[Ak(n)(φχ1A)−φχ1A]χ1A1||p,ω.

Since *φχ*_1_^*A*^ is a continuous function on [−*A*, *A*], for any given *ε*′ > 0, there exists a *δ* > 0 such that

(25) $$\begin{array}{c} \left|\varphi \left(t\right)\chi_{1}^{A}\left(t\right)-\varphi \left(x\right)\chi_{1}^{A}\left(x\right)\right|<\varepsilon^{\prime }+2M_{\varphi }\frac{{\left(t-x\right)}^{2}}{\delta^{2}}. \end{array}$$

Furthermore, this inequality also holds in the case that *t* ∈ [−*A*, *A*] and that *x* ∈ [−*A*_1_, −*A*) ∪ (*A*, *A*_1_] since *φ*(*x*)*χ*_1_^*A*^(*x*) = 0 and *φ*(*t*)*χ*_1_^*A*^(*t*) are continuous. So, we have

(26) sup⁡n||[Ak(n)(φχ1A)−φχ1A]χ1A1||p,ω ≤sup⁡n||[Ak(n)(|φ(t)χ1A(t)−φ(x)χ1A(x)|;x)]χ1A1(x)||p,ω  +sup⁡n||φ(x)χ1A(x)(Ak(n)(1;x)−1)||p,ω ≤(ε′+2Mφδ2A2+Mφ)sup⁡n||Ak(n)(1;x)−1||p,ω+ε′  +4Mφδ2Asup⁡n||Ak(n)(t;x)−x||p,ω  +2Mφδ2sup⁡n||Ak(n)(t2;x)−x2||p,ω.

Using (24) and (26), we can write

(27) sup⁡n In′   ≤3ε′+(ε′+2Mφδ2A2+2Mφ)sup⁡n||Ak(n)(1;x)−1||p,ω  +4Mφδ2Asup⁡n||Ak(n)(t;x)−x||p,ω  +2Mφδ2sup⁡n||Ak(n)(t2;x)−x2||p,ω.

Then we obtain the following equality for (14):

(28) sup⁡n||Ak(n)f−f||p,ω ≤3ε′+(K+1)ε+C{sup⁡n||Ak(n)(1;x)−1||p,ω+sup⁡n||Ak(n)(t;x)−x||p,ω+sup⁡n||Ak(n)(t2;x)−x2||p,ω},

where *C* : = max⁡{*ε*′ + (2*M*_*φ*_/*δ* ^2^)*A* ^2^ + 2*M*_*φ*_, (4*M*_*φ*_/*δ* ^2^)*A*, (2*M*_*φ*_/*δ* ^2^)}. By the hypothesis of theorem and arbitrarity of *ε* and *ε*′, sup⁡_*n*_||*A*_*k*_^(*n*)^ *f*−*f*||_*p*,*ω*_ → 0 as *k* → *∞* which is desired result.

Now we give an example of a sequence of positive linear operators which satisfies the conditions of Theorem 1 in weighted space *L*_*p*,*ω*_(ℝ).

**Example 2**

We choose *ω*(*x*) = *e* ^−*x*^. Note that this selection of *ω* satisfies condition (1). Also note that for 1 ≤ *p* < *∞*

(29) $$\begin{array}{c} L_{p,\omega }\left(\mathbb{R}\right)=\left\{f:\mathbb{R}\to \mathbb{R}:e^{-x}f\left(x\right)\in L_{p}\left(\mathbb{R}\right)\right\}. \end{array}$$

Also *A* ^*n*^ = *C* for each *n* where *C* is the Cesáro matrix; that is,

(30) $$\begin{array}{c} c_{kj}=\{\begin{array}{cc} \frac{1}{k}, & 1\leq j\leq k,\\ 0, & \text{otherwise}. \end{array} \end{array}$$

The Kantorovich variant of the Szász-Mirakyan operators [18] by replacing *f*(*sb*_*k*_/*k*) with an integral mean of *f*(*x*) over the interval [(*s* + 1)*b*_*k*_/*k*, *sb*_*k*_/*k*] is as follows:

(31) Sk(f;x):=kbk∑s=0∞Pk,s(x)∫sbk/k(s+1)bk/kf(t)dt,k∈ℕ,  x∈[0,bk),

where (*b*_*k*_) is a sequence of positive real numbers satisfying the condition

(32) $$\begin{array}{c} \underset{k\to \infty }{\lim}\frac{b_{k}}{k}=0,\;\;\underset{k\to \infty }{\lim}b_{k}=\infty ,\\ P_{k,s}\left(x\right):=e^{-kx/b_{k}}\frac{{\left(kx\right)}^{s}}{s!b_{k}^{s}},\;s=0,1,2,\ldots . \end{array}$$

It is known that

(33) $$\begin{array}{c} S_{k}\left(1;x\right)=1,\;\;S_{k}\left(t;x\right)=x+\frac{b_{k}}{2k},\\ S_{k}\left(t^{2};x\right)=x^{2}+\frac{2b_{k}}{k}x+\frac{b_{k}^{2}}{3k^{2}}. \end{array}$$

Furthermore by simple calculations, we obtain

(34) sup⁡n||Ak(n)(1;x)−1||p,ω=0,sup⁡n||Ak(n)(t;x)−x||p,ω=12k||1||p,ω∑j=1kbjj,sup⁡n||Ak(n)(t2;x)−x2||p,ω ≤2k||x||p,ω∑j=1kbjj+13k||1||p,ω∑j=1kbj2j2.

Also,

(35) sup⁡n,k||Ak(n)||Lp,ω→Lp,ω =sup⁡n,ksup⁡||f||p,ω=1||Ak(n)(f;x)||p,ω<∞.

Hence, conditions (10), (11) are provided which means that for any function *f* ∈ *L*_*p*,*ω*_(ℝ), we have

(36) $$\begin{array}{c} \underset{k}{\lim}\;\underset{n}{\sup}{||A_{k}^{\left(n\right)}f-f||}_{p,\omega }=0. \end{array}$$

Also, analogue of Theorem 1 for the space of function of several variables can be obtained. Now, we establish this theorem. For the sake of convenient notation, we present our second results on *L*_*p*,*Ω*_(ℝ^*m*^), *m* ∈ *ℕ*, instead of *L*_*p*,*Ω*_(ℝ^*n*^) to avoid any confusion about the indices of *𝒜* = {*A* ^*n*^}.

**Theorem 3**

Let *𝒜* = {*A* ^*n*^} be a sequence of infinite matrices with nonnegative real entries and let {*L*_*j*_} be a sequence of positive linear operators from *L*_*p*,*Ω*_(ℝ^*m*^) into *L*_*p*,*Ω*_(ℝ^*m*^). Assume that

(37) $$\begin{array}{c} \underset{n,k}{\sup}{||A_{k}^{\left(n\right)}||}_{L_{p,\Omega }\to L_{p,\Omega }}<\infty . \end{array}$$

If

(38) lim⁡k sup⁡n||Ak(n)(1;x)−1||p,Ω=0,lim⁡k sup⁡n||Ak(n)(ti;x)−xi||p,Ω=0, i=1,2,…,m,lim⁡k sup⁡n||Ak(n)(|t|2;x)−|x|2||p,Ω=0,

then for any function *f* ∈ *L*_*p*,*Ω*_(ℝ^*m*^), one has

(39) $$\begin{array}{c} \underset{k}{\lim}\;\underset{n}{\sup}{||A_{k}^{\left(n\right)}f-f||}_{p,\Omega }=0. \end{array}$$

**Proof**

Considering the characteristic function *χ*_1_^*A*^ of the ball |*x*| ≤ *A* and *χ*_2_^*A*^(*t*) = 1 − *χ*_1_^*A*^(*t*), it is possible to choose a sufficient large *A* such that

(40) $$\begin{array}{c} {||f\chi_{2}^{A}\left(t\right)||}_{p,\Omega }<\varepsilon . \end{array}$$

On the other hand, by condition (37) there exists a positive constant *K* such that ||*A*_*k*_^(*n*)^||_*p*,*Ω*_ ≤ *K*, and so, for given *ε*′ > 0, there exists a continuous function *θ* on |*x*| ≤ *A* satisfying the condition *θ*(*x*) = 0 for |*x*| > *A* such that

(41) $$\begin{array}{c} {||\left(f-\theta \right)\chi_{1}^{A}||}_{p,\Omega }<\frac{\varepsilon^{\prime }}{\left(K+1\right){\left(\max_{|t|\leq A}\Omega \left(t\right)\right)}^{1/p}}. \end{array}$$

Keeping in mind the fact that the series (9) is a convergence for each *k*, *n*, and *f*, and using the linearity of the operators *L*_*j*_, which means the linearity of *A*_*k*_^(*n*)^, we get

(42) sup⁡n||Ak(n)f−f||p,Ω≤sup⁡n||Ak(n)(χ1Aθ)−χ1Aθ||p,Ω +(K+1)ε+ε′.

Let *A*_1_ > *A*, so we also have

(43) sup⁡n||Ak(n)(χ1Aθ)−χ1Aθ||p,Ω ≤sup⁡n||[Ak(n)(χ1Aθ)−χ1Aθ]χ1A1||p,Ω  +Mθsup⁡n||Ak(n)(1)−1||p,Ω  +Mθ||χ2A1||p,Ω,

where *M*_*θ*_ : = max⁡_*t*∈ℝ^*m*^_|*θ*(*t*)|*χ*_1_^*A*^(*t*). Furthermore, we can choose *A*_1_ such that ||*χ*_2_^*A*_1_^||_*p*,*Ω*_ < *ε*′/*M*_*θ*_, and for sufficiently large *k*, we estimate sup⁡_*n*_||*A*_*k*_^(*n*)^(1)−1||_*p*,*Ω*_ < *ε*′/*M*_*θ*_. Using these estimations in (42), we obtain

(44) sup⁡n||Ak(n)f−f||p,Ω≤sup⁡n||[Ak(n)(χ1Aθ)−χ1Aθ]χ1A1||p,Ω +(K+1)ε+3ε′.

Since

(45) $$\begin{array}{c} \left|\chi_{1}^{A}\left(t\right)\theta \left(t\right)-\chi_{1}^{A}\left(x\right)\theta \left(x\right)\right|<\varepsilon^{\prime }+2M_{\theta }\frac{{\left|t-x\right|}^{2}}{\delta^{2}}, \end{array}$$

we can write

(46) sup⁡n||Ak(n)f−f||p,Ω ≤(K+1)ε+4ε′K||Ω||11/P  +2Mθ(1+A)2δ2{sup⁡n||Ak(n)(|t|2;x)−|x|2||p,Ω+∑l=0msup⁡n||Ak(n)(ti;x)−xi||p,Ω+sup⁡n||Ak(n)(1)−1||p,Ω}.

Using the conditions of theorem, we have

(47) sup⁡n||Ak(n)f−f||p,Ω ≤(K+1)ε+4ε′K||Ω||11/P+2Mθ(1+A)2δ2  ×[δ2ε1∗2Mθ(1+A)2+mδ2ε2∗2Mθ(1+A)2+δ2ε3∗2Mθ(1+A)2] =(K+1)ε+4ε′K||Ω||11/P+ε1∗+mε2∗+ε3∗,

which means that

(48) $$\begin{array}{c} \underset{k}{\lim}\;\underset{n}{\sup}{||A_{k}^{\left(n\right)}f-f||}_{p,\Omega }=0. \end{array}$$

Now we give the following example.

**Example 4**

We choose *Ω*(*x*, *y*) = *e* ^−*x*−*y*^. Note that this selection of *Ω* satisfies condition (3). Also note that, for 1 ≤ *p* < *∞*,

(49) $$\begin{array}{c} L_{p,\Omega }\left({\mathbb{R}}^{2}\right)=\left\{f:{\mathbb{R}}^{2}\to \mathbb{R}:\Omega \left(x,y\right)f\left(x\right)\in L_{p}\left({\mathbb{R}}^{2}\right)\right\}. \end{array}$$

Also *A* ^*n*^ = *C* for each *n* where *C* is the Cesáro matrix, that is;

(50) $$\begin{array}{c} c_{kj}=\{\begin{array}{cc} \frac{1}{k}, & 1\leq j\leq k,\\ 0 & \text{otherwise}. \end{array} \end{array}$$

The Kantorovich variant of the Szász-Mirakyan operators [18] by replacing *f*(*tb*_*k*_/*k*, *sb*_*k*_/*k*) with an integral mean of *f*(*x*, *y*) over the interval [(*t* + 1)*b*_*k*_/*k*, *tb*_*k*_/*k*] × [(*s* + 1)*b*_*k*_/*k*, *sb*_*k*_/*k*] is as follows:

(51) Sk(f;x,y):=k2bk2∑t=0∞∑s=0∞Pk,t,s(x,y)×∫tbk/k(t+1)bk/k∫sbk/k(s+1)bk/kf(u,v)du dv,k∈ℕ,  x,y∈[0,bk),

where (*b*_*k*_) is a sequence of positive real numbers satisfying the condition

(52) lim⁡k→∞bkk=0,  lim⁡k→∞bk=∞,Pk,t,s(x,y)≔e−(k(x+y)/bk)(kx)t(ky)st!s!bkt+s,t,s=0,1,2,….

It is known that

(53) Sk(1;x,y)=1,Sk(u;x,y)=x+bk2k,Sk(v;x,y)=y+bk2k,Sk(u2+v2;x,y) =x2+y2+2bkk(x+y)+2bk23k2.

Furthermore we obtain

(54) sup⁡n||Ak(n)(1;x,y)−1||p,Ω=0,sup⁡n||Ak(n)(u;x,y)−x||p,Ω=12k||1||p,Ω∑j=1kbjj,sup⁡n||Ak(n)(v;x,y)−y||p,Ω=12k||1||p,Ω∑j=1kbjj,sup⁡n||Ak(n)(u2+v2;x,y)−(x2+y2)||p,Ω ≤2k||x+y||p,Ω∑j=1kbjj+23k||1||p,Ω∑j=1kbj2j2.

Also,

(55) sup⁡n,k||Ak(n)||Lp,Ω→Lp,Ω =sup⁡n,ksup⁡||f||p,Ω=1||Ak(n)(f;x,y)||p,Ω<∞.

Hence, conditions (10) and (11) are provided which means that for any function *f* ∈ *L*_*p*,*Ω*_(ℝ^2^), we have

(56) $$\begin{array}{c} \underset{k}{\lim}\underset{n}{\sup}{||A_{k}^{\left(n\right)}f-f||}_{p,\Omega }=0. \end{array}$$
